# Establishment and Characterization of a Telomerase-Immortalized Sheep Trophoblast Cell Line

**DOI:** 10.1155/2016/5808575

**Published:** 2016-02-21

**Authors:** Yufei Zhang, Jing Shi, Shuying Liu

**Affiliations:** ^1^College of Veterinary Medicine, Inner Mongolia Agricultural University, Hohhot 010018, China; ^2^Key Laboratory of Clinical Diagnosis and Treatment Technology in Animal Disease, Ministry of Agriculture, Hohhot 010018, China

## Abstract

The primary sheep trophoblast cells (STCs) have a finite lifespan in culture. This feature limits the scope for long-term* in vitro* studies with STCs. This study was an attempt to establish and characterize a telomerase-immortalized sheep trophoblast cell line. STCs were isolated and purified by using Percoll and specific immunoaffinity purification, respectively. The purified STCs were transfected with a plasmid carrying sequences of human telomerase reverse transcriptase (hTERT) to create immortalized sheep trophoblast cell line (hTERT-STCs). hTERT-STCs showed a stable expression of hTERT gene, serially passaged for a year, and showed active proliferation without signs of senescence. Cytokeratin 7 (CK-7), secreted human chorionic gonadotrophin subunit *β* (CG-*β*), placental lactogen (PL), and endogenous jaagsiekte sheep retrovirus (enJSRV) envelope genes were expressed in hTERT-STCs. Transwell cell invasion assay indicated that hTERT-STCs still possessed the same invasive characteristics as normal primary sheep trophoblast cells. hTERT-STCs could not grow in soft agar and did not develop into tumors in nude mice. In this study, we established a strain of immortalized sheep trophoblast cell line which could be gainfully employed in the future as an experimental model to study trophoblast cells with secretory function, invasive features, and probable biological function of enJSRV envelope genes.

## 1. Introduction

Mammalian placentae are unique organs serving as the interface between the fetal and maternal tissues. Although placentae of different animals are dissimilar in shapes, sizes, and organizational structure, all share the same functionality; for example, the placentae have a fundamental role in the exchange of nutrients, ions, water, respiratory gases, vitamins, nutrients, and other molecules which are necessary for fetal metabolism and development. In addition, the placenta produces hormones and also acts as a barrier that protects the fetus from insult from the maternal immune system [1].

The functional cell type of the placenta is the trophoblast cell. There are some differences between the type and role of trophoblast cells among different species. Human placental trophoblasts include cytotrophoblast (CTB), syncytiotrophoblast (STB), and extravillous trophoblast (EVT) [2]. The multinucleated syncytiotrophoblasts covered on the surface of villi are formed by differentiation and fusion of cytotrophoblasts [3, 4]. The multinucleated syncytiotrophoblasts secrete hormones such as human chorionic gonadotrophin (HCG) and placental prolactin. These hormones enter the maternal circulation and play an important role in the maintenance of pregnancy and immune response [4, 5]. Extravillous cytotrophoblasts exhibit strong extended invasiveness and erode and invade the endometrium until maternal spiral arteries are able to anchor the conceptus in uterus, while the CTB represents the medium for the nutrient and gas exchange vital for the growth of the fetus [6, 7]. Histologically, the sheep placenta is classified as cotyledonary placenta or multiplex type of placenta. Trophoblast cells differentiate into four subsets, that is, mononuclear trophoblast cells (MTCs), binucleate cells (BNCs), trinucleate cells (TNCs), and multinucleated syncytiotrophoblasts [6, 8]. The BNCs differentiate from the MTC through mitosis and polyploidy formation which involves consecutive nuclear divisions without cytokinesis [9, 10]. Some of the BNCs merge with the endometrial epithelial cells of the uterus and evolve as TNCs. Other BNCs fuse with the trinucleate cells to form multinucleated syncytial plaques with 20–25 nuclei [9]. Cotyledons, the specialized structure formed between the multinucleated syncytial plaques and BNC, take shape as placentome with maternal endometrium through reciprocal chiasmata [9, 11]. Hemotrophic nutrition is passed through the placentomes to the fetus [9]. Other functions of multinucleated syncytial plaques and BNC include the production of important proteins and hormones, such as placental prolactin, chorionic gonadotrophin, pregnancy associated glycoprotein, and progesterone [9].

The trophoblast cell lesions are often involved in the causation of obstetric disorders including fetal growth restriction, preeclampsia, and even abortion [6, 12, 13]. Therefore, trophoblast cell lines generated from normal cells represent an invaluable vehicle for research on trophoblast cell biology, immunology, endocrine function, placental development, and mechanisms of infectious and noninfectious diseases [14]. However, the isolated primary trophoblast cells rapidly lose the ability to proliferate, to differentiate, and to secrete hormones, which greatly limits the scope of its use in* in vitro* studies. Establishment of a cell line that can overcome this limitation is a key research imperative.

Cultured normal human cells have a finite lifespan due to replicative senescence, which is associated with progressive shortening of cell telomeres [6, 15, 16]. Telomeres found at the ends of chromosomes in eukaryotes have been shown to protect the chromosome ends and maintain cell immortality [16, 17]. By introducing exogenous telomerase reverse transcriptase (hTERT) gene, cells appeared to acquire the ability for unlimited proliferation through the activation of telomerase [18, 19]. Studies have shown that the introduction of hTERT gene enables establishment of immortalized cell line which retains the original characteristics of the normal cells [6, 20, 21].

In this study, we sought to establish a stable sheep trophoblast cell line expressing exogenous hTERT gene and profiled its phenotype and functionality.

## 2. Materials and Methods

### 2.1. Isolation, Purification, and Culture of Sheep Trophoblast Cells

Pregnant Mongolian sheep uteri (45–60 days of pregnancy) provided by the Hohhot slaughterhouse were immediately transferred to the laboratory in a thermal container with a heat preservation vessel containing sterilized saline at 37°C. The phase of pregnancy was estimated by measuring the fetal crown rump length [6]. The primary sheep trophoblast cells (STCs) were separated from the tissue samples and cultured as described by Petroff et al. [22] with some modifications. In brief, the uterus was cleaned with 70% ethanol and dissected in the sterile console, and the cotyledon was mechanically separated with tweezers and placed in a sterile Petri dish 10 cm in diameter. The cotyledons were meticulously minced and dissociated in 100 mL Hank's balanced salt solution (HBSS) with 25 mmol HEPES, 0.2 mg/mL DNaseI (Sigma, St. Louis, MO, USA), and 0.25% trypsin (Invitrogen, Carlsbad, CA, USA) for 30 min at 37°C in a rotating water-bath shaker (150 rpm). The dispersed cells were isolated by 200 *μ*m mesh stainless steel screens and kept in 15% fetal bovine serum (FBS; Gibco, Grand Island, NY, USA). The filtrate was centrifuged at 1000 g for 10 min and resuspended in serum-free DMEM/F12 medium (Gibco, Grand Island, NY, USA). The cell suspension was transferred to a test tube containing 30% and 50% Percoll gradient and centrifuged continuously with a swinging bucket rotor at 1200 g for 20 min at room temperature (RT). The upper diffuse band containing the STCs was aspirated with Pasteur pipette. The cells were washed by a fourfold volume of nonserum culture medium and centrifuged at 1000 g for 5 min and suspended with 1 mL cell separation buffer (0.5% BSA, 0.08% EDTA pH 7.2, PBS, and vacuum filtration sterilization as well as liquid within gas). Cells (2 × 10^8^ cells/mL) were incubated with 40–60 *μ*g/mL anti-human HLA-ABC (Miltenyi Biotec, Bergisch Gladbach, Germany) for 30 min at 4°C. The cells were incubated with goat anti-mouse IgG microbeads (Miltenyi Biotec, Bergisch Gladbach, Germany) and then purified by immune negative selection. After immunodepletion, the cells were centrifuged at 400 g and resuspended in complete DMEM/F12 medium within 15% fetal bovine serum. The purified cells were plated onto applicable culture vessels with culture medium at a minimum of 2 × 10^5^ cells/cm^2^ and incubated in an atmosphere of 5% CO_2_ at 37°C.

### 2.2. Transfections and Establishment of Cell Line

The primary sheep trophoblast cells were treated with 0.25% trypsin to obtain the single cells. The cells were then washed with preheated Opti-MEM Medium twice, and their density was adjusted to 1 × 10^7^/mL. After a gentle mix, the mixture of 100 *μ*L cell suspension (approximately 1 × 10^6^ cells) and 10 *μ*g pCI-neo-hTERT plasmid DNA was placed in 2 mm gap electroporation cuvettes (BTX, Holliston, MA, USA) and subjected to a pulse of 150 V and 5 ms pulse length by an electroporator (NEPA21; Tokiwa Science, Tokyo) and then seeded into preheated 100 mm dish containing 15 mL complete DMEM/F12 medium. The transfected cells were plated in medium containing 500 *μ*g/mL G418 (Sigma, St. Louis, MO, USA) for two to three weeks 48 h after transfection. The hTERT-transfected sheep trophoblast cell (hTERT-STCs) clones were cultured in complete DMEM/F12 medium supplemented with EGF (Sigma, St. Louis, MO, USA) and 300 *μ*g/mL of G418 [6].

### 2.3. Reverse Transcription Polymerase Chain Reaction for cDNA Synthesis

Total RNA from STCs and hTERT-STCs was isolated by using RNAprep Pure Micro Kit (TIANGEN, Beijing, China) according to the manufacturer's instructions. Reverse transcriptase polymerase chain reaction (RT-PCR) was performed using One-Step RT-PCR Kit method (TaKaRa, Dalian, China). The primers of hTERT, GAPDH, endogenous jaagsiekte sheep retrovirus envelope gene (enJSRV-*env*), and* syncytin-Rum1 *were designed by mRNA sequences of [NM_198253.2], [NM_001190390.1], [AF153615], and [JX412969.1] to obtain the respective cDNAs. PCR was performed using particular conditions for the following genes: hTERT, GAPDH, enJSRV-*env, *and* syncytin-Rum1.* The PCR conditions used during reactions are mentioned in Table 1. Following the PCR reaction, products were electrophoresed by 1% agarose gel electrophoresis and stained with ethidium bromide.

### 2.4. Western Blot Analysis

The day before transfection, 1.0 × 10^5^ cells of primary STCs and hTERT-STCs were, respectively, plated in 60 mm Petri dish and total proteins were extracted from 24 h culture by using the M-PER Mammalian Kit (Thermo Fisher Scientific, Beijing, China). Protein samples were normalized with Bradford reagent (Bio-Rad, Beijing, China). After separation by SDS-PAGE electrophoresis, proteins were transferred to a PVDF membrane using the semidry method with constant voltage of 25 V for 15 min. The membrane was blocked with 5% nonfat dry milk for 1 h at RT and then incubated with rabbit polyclonal anti-telomerase reverse transcriptase (1 : 500; Abcam, Cambridge, MA, USA), overnight at 4°C. Blots were washed in TBST thrice for 5 min each. The membrane was then incubated with goat anti-rabbit secondary antibody with HRP conjugation (1 : 2000; Abcam, Cambridge, MA, USA) for 2 h at 37°C. After three washes with TBST, the membrane blots were detected by chemiluminescence. GAPDH were used as internal controls.

### 2.5. Immunofluorescence

5 × 10^3^ cells were seeded onto chamber slides until 50% confluency was achieved. They were washed with PBS and fixed with 4% paraformaldehyde at RT for 15 min. After permeabilization with 0.1% Triton X-100 in PBS for 5 min at RT, the cells were blocked with 10% normal goat serum for 1 h. The cells were incubated with rabbit monoclonal anti-cytokeratin 7 (CK-7) antibodies (1 : 200; Thermo Fisher Scientific, Beijing, China) containing 10% normal goat serum for 14 h at 4°C. With a subsequent wash, the cells were incubated with goat anti-rabbit-Texas Red labelled antibodies (1 : 100; Thermo Fisher Scientific, Beijing, China) for 1 h at 37°C and were mounted with cover slips and observed under a Zeiss Axio Observer microscope (Carl Zeiss, Oberkochen, Germany) after staining with 0.5 *μ*g/mL 4-6-diamidino-2-phenylindole (DAPI) for 10 min.

### 2.6. Hormone Assay

The assay method described by Dong et al. was used for assessing the secretion of chorionic gonadotrophin and placental lactogen [6].

### 2.7. Cell Invasion Assay

Transwell membranes (8.0 *μ*m pore size, Coring, NY, USA) coated with Matrigel on upper and bottom chambers were dried at 4°C for 1 h. The upper chamber was incubated with 300 *μ*L of preheated serum-free medium for 30 min at RT for matrix rehydration. Primary STCs and hTERT-STCs (3 × 10^5^) were plated in the upper chamber in serum-free Opti-MEM I Medium, respectively. The lower chambers were filled with 500 *μ*L complete DMEM/F12 medium. Following an incubation for 48 h, the culture medium was discarded and washed with PBS. The cells on Matrigel and upper chamber were wiped with a cotton swab. The migrated cells were fixed with 90% ethanol for 30 min at RT, stained with Giemsa, and examined under a light microscope.

### 2.8. Soft Agar Assay and Test of Tumorigenicity

Six-well plates were filled with two mL of 0.66% noble agar as a bottom layer. Each of the 3000 primary STCs and hTERT-STCs cells, respectively, was blended with 0.35% soft agar and plated onto wells with 5% CO_2_ and cultured at 37°C for 14 days. HeLa cells served as positive control, and the results were observed under an inverted microscope.

Cells in the logarithmic growth phase were collected and subjected to trypsinization, washed with PBS thrice, and suspended in a serum-free medium and the cell concentration was adjusted to 1 × 10^7^/mL by cell counting. 0.2 mL cell suspension was subcutaneously injected into the right flanks of nude mice. After two months, the mice were decapitated and dissected for subcutaneous tumor nodules. These were paraffin embedded and subjected to hematoxylin and eosin (H&E) staining.

### 2.9. Statistical Tests

Data are presented as mean ± standard deviation (SD). Statistical analyses were conducted using SAS 8.1 (SAS Institute, Cary, NC, USA) to determine intergroup differences in secretion of hormones; *P* < 0.05 was considered statistically significant.

## 3. Results

### 3.1. Morphological Characteristics of STCs and hTERT-STCs

**Figure 1:**
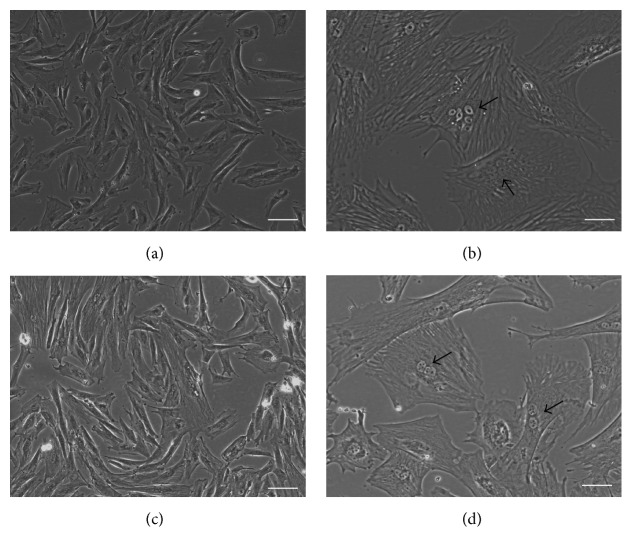
Primary sheep trophoblast cells and immortalized sheep trophoblast cells under phase contrast microscopy. (a) Primary STCs at passage 2; (b) multinucleated syncytiotrophoblast from primary STCs; (c) hTERT-STCs at passage 50; and (d) binucleate trophoblast cells from hTERT-STCs (scale bars, 50 *μ*m).* hTERT-STCs: human telomerase reverse transcriptase-sheep trophoblast cell line*.

The primary sheep trophoblast cells (STCs) obtained from pregnant Mongolian sheep (45–60 days of pregnancy) were mainly mononuclear cells that showed epithelial cell-like growth and morphological diversity, with oval nuclei (Figure 1(a)). On subculture of cells, intercellular fusion formed binucleate trophoblast cells, multinucleated syncytium (Figure 1(b)). After trypsinization and subculturing of sheep trophoblast cells, adherent growth was observable within 4 h. However, with the increase of trophoblast cell passage number, cell proliferation was visibly decreased and had stopped growing by the 7th generation, with a large number of cells dead on account of senescence.

The STCs were transfected with pCI-neo plasmid containing the cDNA for hTERT for conversion to an immortalized sheep trophoblast cell line referred to as hTERT-STCs. The morphology of hTERT-STCs cells remained similar to primary STCs during one year of cultivation (Figure 1(c)). hTERT-STCs could also form binucleate trophoblast cells and multinucleated syncytium through intercellular fusion. Due to lesser number of multinucleated syncytium cells, we gave priority to binucleate trophoblast cells (Figure 1(d)).

### 3.2. hTERT Expression in hTERT-STCs

**Figure 2:**
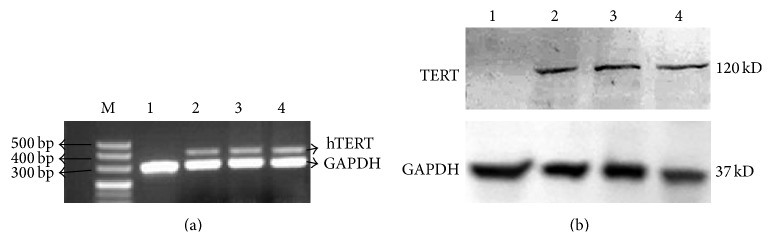
Retention of telomerase expression in hTERT-STCs. (a) Comparison of* hTERT* gene expression between hTERT-STCs and STCs by RT-PCR. M was the DL 500 DNA makers. Lane 1 was primary STCs; lane 2 was hTERT-STCs at passage 20; lane 3 was hTERT-STCs at passage 50; and lane 4 was HeLa cells (positive control). (b) Comparison of hTERT protein expression between hTERT-STCs and STCs by Western blot. Lanes 1–4 are the same as mentioned in (a).* hTERT-STCs: human telomerase reverse transcriptase-sheep trophoblast cell line. RT-PCR: reverse transcriptase polymerase chain reaction*.

Total RNA and total protein of primary STCs, 30th and 50th generation hTERT-STCs cells, and HeLa cells were extracted and subjected to RT-PCR and Western blot analysis. PCR product was confirmed by 1% agarose gel electrophoresis analysis. The results showed that the total RNA of hTERT-STCs and HeLa cells showed expression of amplified 400 bp hTERT band, while no such band was observed in the primary STCs (Figure 2(a)). This suggests that the hTERT-STCs cells had a stable expression of the mRNA of* hTERT* gene. Similar results were obtained by Western blot assay, where the hTERT protein (120 kD) was expressed in hTERT-STCs and HeLa cells, but not in primary STCs (Figure 2(b)). These results indicate that the immortalized hTERT-STCs obtained by this method retained the ability to proliferate* in vitro* and were amenable to culture in the longer term.

### 3.3. Phenotypic Characteristics of hTERT-STCs

**Figure 3:**
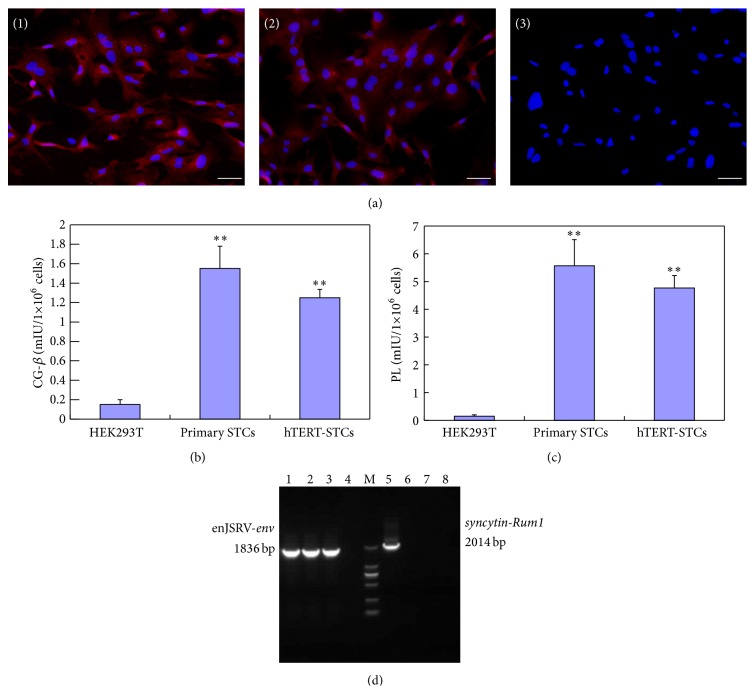
The biological characteristics of immortalized STCs were found to be similar to that of primary STCs. (a) Immunofluorescence staining of primary STCs for cell keratin 7 expression (1) and hTERT-STCs at passage 50 (2) was positive. Immunofluorescence staining of hTERT-STCs for PBS was (3) (scale bars, 50 *μ*m). (b) The secretion level of primary STCs and hTERT-STCs at passage 50 CG-*β*, HEK293T cells (negative control) (^*∗∗*^ *P* < 0.01). (c) The secretion level of primary STCs and hTERT-STCs at passage 50 PL, HEK293T cells (negative control) (^*∗∗*^ *P* < 0.01). (d) The expression of enJSRV-env and syncytin-Rum1 was detected by RT-PCR. Lane 1 was Mongolian sheep genomic DNA; lane 2 was primary STCs; lane 3 was hTERT-STCs at passage 50; and lane 4 was negative control. Lanes 5–8 are the same as in lanes 1–4.* hTERT-STCs: human telomerase reverse transcriptase-sheep trophoblast cell line*.* RT-PCR: reverse transcriptase polymerase chain reaction*.

The most widely used phenotypic markers of trophoblast cell lines are cytokeratins, placental lactogen (PL), chorionic gonadotrophin (CG), and leukocyte antigen class I molecules [14]. The methods described in this research summarize the enzymatic dispersion, density gradient centrifugation, and leukocyte antigen class I (LAC-I) depletion of trophoblast cells. There are other placental cell types which express LAC-I molecules, except for the trophoblast cells. Thus, using the latter method to deplete these types of hybrid cells, we obtained cells which did not express the LAC-I molecules. Immunofluorescence staining for cytokeratin 7 showed that the primary STCs purity was >97% (Figure 3(a) (1)) and 100% pure hTERT-STCs (Figure 3(a) (2)).

The hormone analysis of cell culture revealed that both primary STCs and hTERT-STCs secreted CG-*β* and PL, with no significant difference in secretory levels of these hormones (*P* > 0.05) (Figures 3(b) and 3(c)). In the sheep placenta, enJSRV-*env* is expressed in the mononuclear trophectoderm cells of the conceptus and is abundant in the trophoblast giant binucleated cells (BNCs) and multinucleated syncytial plaques of the placentomes [23–25].

The RT-PCR for enJSRV-env and syncytin-Rum1 showed that enJSRV-*env* was detected in the primary STCs and hTERT-STCs, but syncytin-Rum1 was detected only in the sheep genome (Figure 3(d)).

### 3.4. *In Vitro* Invasion Assay and Proliferation Analysis of Primary STCs and hTERT-STCs

**Figure 4:**
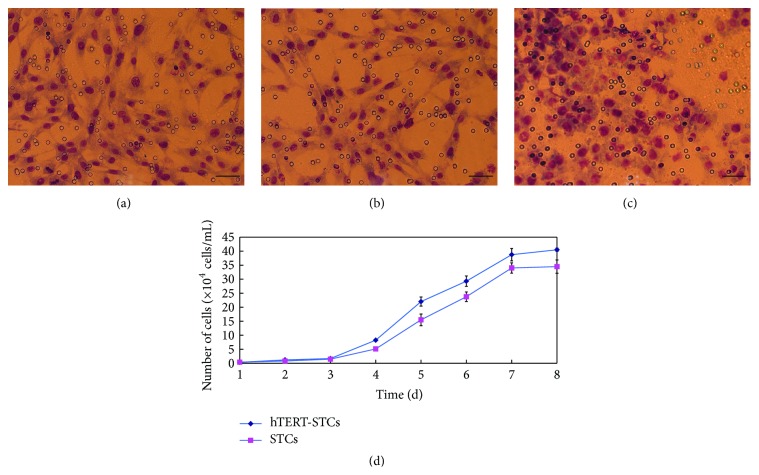
Growth characteristics and invasiveness of primary STCs and immortalized STCs. Primary STCs (a) and hTERT-STCs at passage 50 (b) are seen invading the lower portion of the chamber on Giemsa staining. HeLa cells (c) served as a positive control (scale bars, 50 *μ*m). (d) Growth curves of primary STCs and hTERT-GTCs at passage 50.* hTERT-STCs: human telomerase reverse transcriptase-sheep trophoblast cell line*.

The invasive capacity of trophoblast cells is an essential prerequisite for the development of mammalian placenta. We studied the invasive characteristics of primary STCs and hTERT-STCs* in vitro* by Transwell invasion assay; the results showed that both primary STCs (Figure 4(a)) and hTERT-STCs (Figure 4(b)) exhibited invasive ability, indicating that the latter had inherited the invasive ability of the former. HeLa cells (Figure 4(c)) served as a positive control.

Cell counting method was used to test the proliferation ability of primary STCs and hTERT-STCs cells, and the results when plotted as curves (Figure 4(d)) demonstrated two different kinds of cell proliferation patterns, which tended to increase with time. No significant difference was observed between the proliferation abilities of primary STCs and hTERT-STCs (*P* > 0.05).

### 3.5. Nonmalignant Transformation of hTERT-STCs* In Vitro* and* In Vivo*

**Figure 5:**
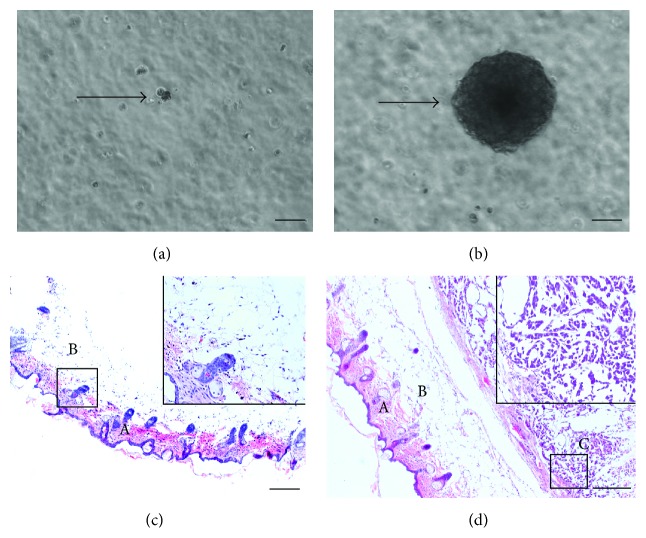
HTERT-STCs did not undergo malignant transformation. (a) hTERT-STCs did not form cell colonies after > 2 weeks of culture (scale bar, 50 *μ*m). (b) HeLa cells which served as positive control formed cell colonies in the same cultivation time (scale bar, 50 *μ*m). (c) The organizational structure of nude mice that were injected with hTERT-STCs showing no abnormal cell proliferation at the vaccination site after 2 months. Epidermis and dermis of the skin of the injection site in nude mice (A). Normal subcutaneous connective tissue (B) (scale bar, 25 *μ*m). The selected area is enlarged 5 times in small panels. (d) The organizational structure of nude mice that were injected with HeLa cells, showing a large number of abnormally proliferating tumor cells at the vaccination site after 3 weeks. Epidermis and dermis of the skin of the injection site in nude mice (A). Normal subcutaneous connective tissue (B). Subcutaneous connective tissue with a large number of proliferating tumor cells (C) (scale bar, 25 *μ*m). The selected area is enlarged 5 times in small panel.* hTERT-STCs: human telomerase reverse transcriptase-sheep trophoblast cell line*.

Soft agar-based clonogenic assays are commonly employed to detect transformed cells. Soft agar-based clonogenic assays were used to detect malignant transformation of hTERT-STCs. We observed that after 14 days of incubation hTERT-STCs could not form colonies (Figure 5(a)), as compared to positive control HeLa (Figure 5(b)), which formed clones greater than 100 *μ*m diameter. This suggests that the hTERT-STCs had not acquired the ability of anchorage-independent growth.

The tumorigenicity of the immortalized sheep trophoblast cell line was then studied* in vivo* by subcutaneous injection of 1 × 10^6^ hTERT-STCs cells in the flank of nude mice. No evidence of tumor formation was observed in these mice after 2 months. Histological examination revealed normal loose connective tissue below the injection sites and abnormally hyperplastic cells (Figure 5(c)). Nude mice injected with HeLa cells developed a localized mass at 3 weeks, which gradually increased, with a large number of abnormally proliferating tumor cells as evidenced by histological dysplasia (Figure 5(d)). These data indicate that the hTERT-STCs had become immortal cells but had acquired no other transformation characteristics, rendering them virtually akin to normal cells.

## 4. Discussion

Placenta is composed of trophoblast cells, macrophages, fibroblasts, stromal cells, mesenchymal stem cells, and some other varieties of cells. Among these, the trophoblast cells are the most common ones. Despite some shortcomings, enzyme digestion and tissue block are the most commonly used methods for isolation of trophoblast cells for primary culture. In this study, we used 30% and 50% concentration of Percoll to separate trophoblast cells, which removed a large number of blood cells, and some tissue debris fibroblasts. Finally, we used MACS filter to remove fibroblast cells, stromal cells, and mesenchymal stem cells. The immunofluorescence of purified sheep trophoblast cells revealed that 97% of the cells expressed specific marker such as CK7. This implied that the purified ovine trophoblast cells could be used for further studies.

Primary STCs retain most of their physical characteristics* in vivo*, thus mirroring the status and functionality of these cells under normal physiological conditions, making them well suited for use in studies on cell biology. However, the number of each separation and the culture time are limited, and the STCs last for about seven generations before stoppage of proliferation. The requirement for repeated separation and culture of primary STCs not only is time consuming, but is also affected by availability of placenta. Moreover, there may be differences between different batches of cells by virtue of being in different physiological states. To overcome this limitation, there was a need to establish immortal STCs cell lines* in vitro*.

Immortalization is often achieved by using some viral genes, such as simian virus 40 large T antigen (*SV40 Tag*), human papilloma virus E6/E7 genes, and Epstein-Barr virus (EBV) of membrane protein genes, and by using nonviral hTERT gene. By expressing hTERT in human somatic cells, telomerase activity could be reconstructed and a variety of cell lines are established. The sequence and structure of mammalian telomerase RNA were highly conserved, and nucleotide sequence was the same as that of the reverse template. It follows that, in other mammalian cells, expression of hTERT could be combined with telomerase RNA component to reconstruct the functionality of telomerase. Many immortalized mammalian cell lines have been established using this method [6, 26].

In this study, hTERT gene was transduced onto STCs to obtain stable cell line hTERT-STCs which can be maintained for 50 generations. We observed continuous expression of TERT gene as indicated by RT-PCR and Western blotting results. Thus the cell line appeared to conserve the telomerase activity. These cells showed monolayer form of growth typical of cobblestone morphology, and cells were amenable to stable culture. The results of cell growth curve showed that the growth rate of hTERT-STCs was similar to that of primary STCs.

A study comparing the biological characteristics of immortalized and primary sheep trophoblast cells found that the former expressed trophoblast markers such as protein CK7 and demonstrated immortalized STCs with unaltered characteristics of its epithelial origin. At the same time, immortalized sheep trophoblast cells secreted chorionic gonadotrophin (CG) and placental lactogen (PL), although there was no significant difference as compared to primary STCs (*P* > 0.05). Cell invasive studies showed that immortalized sheep trophoblast cells maintained the invasive characteristics of primary STCs. In addition, Cornelis et al. [27] found an envelope gene from the genome of sheep and cattle such that specific expression in the* Ovis aries *and the* Bos taurus* in the trophectoderm cells of the conceptus was termed syncytin-Rum1. Of note, the expression of enJSRV-env gene was detected in immortalized sheep trophoblast cells and primary ovine trophoblast cells, but not in syncytin-Rum1. EnJSRV-env largely expressed in the sheep placenta trophectoderm cells in a temporal fashion that corresponded with critical points in onset of trophoblast giant BNC differentiation and conceptus elongation [9, 23, 28]. Therefore immortalized sheep trophoblast cells provide a unique model to study the biological role of enJSRV-env in the development and differentiation of the sheep placenta. The detection of the transformation ability for immortalized sheep trophoblast cells* in vitro* and* in vivo* indicated that they behaved essentially as normal cells without significant transformation ability. They could not grow in the soft agar, did not exhibit any tumorigenicity, and retained the contact inhibition property. These findings attest to the maintenance of biological attributes of primary STCs in hTERT-STCs.

## 5. Conclusion

In conclusion, we established a strain of immortalized sheep trophoblast cell line which retained the basic biological characteristics of primary ovine trophoblast with no potential for malignant transformation. This cell line could be invaluable as an experimental model to study the functionality of trophoblast cells such as hormone secretion, invasiveness, and probable biological function of enJSRV envelope genes.

## Figures and Tables

**Table 1:** Primers and conditions used in RT-PCR gene expression.

Gene	Sequence (5′-3′)	Cycles	Product size (bp)	Anneal. temp. °C
hTERT		35	408	55
Sense	CGTACATGCGACAGTTCGTG			
Antisense	AGTTCACCACTGTCTTCCGC			

enJSRV-*env*		35	1836	60
Sense	ATGCCGAAGCGCCGCGCTGGATT			
Antisense	TCACGGGTCGTCCCCCGCAGCTC			

*syncytin-Rum1*		35	2014	60
Sense	GATGGAGCTGGGTAAGCGAC			
Antisense	TTGTAGCCACGAGTTCCAGG			

GAPDH		35	300	55
Sense	GTTTGTGATGGGCGTGAACC			
Antisense	CCAGTGAGCTTCCCGTTGAG			

RT-PCR: reverse transcriptase polymerase chain reaction; enJSRV-*env*: endogenous jaagsiekte sheep retrovirus envelope gene; and hTERT: human telomerase reverse transcriptase.
